# Hyperhomocysteinemia Increases Cortical Excitability and Aggravates Mechanical Hyperalgesia and Anxiety in a Nitroglycerine-Induced Migraine Model in Rats

**DOI:** 10.3390/biom12050735

**Published:** 2022-05-23

**Authors:** Elena Gerasimova, Olga Yakovleva, Daniel Enikeev, Ksenia Bogatova, Anton Hermann, Rashid Giniatullin, Guzel Sitdikova

**Affiliations:** 1 Department of Human and Animal Physiology, Institute of Fundamental Medicine and Biology, Kazan Federal University, 18 Kremlevskaya Str., 420008 Kazan, Russia; gerasimova.el.2011@yandex.ru (E.G.); a-olay@yandex.ru (O.Y.); danielveenik@gmail.com (D.E.); kowarik.ru@yandex.ru (K.B.); 2 Department of Neurobiology, Sirius University of Science and Technology, 1 Olympic Ave., 353340 Sochi, Russia; 3 Department of Biosciences, University of Salzburg, 5020 Salzburg, Austria; anton.hermann@plus.ac.at; 4 A.I Virtanen Institute, University of Eastern Finland, FI-70211 Kuopio, Finland; rashid.giniatullin@uef.fi

**Keywords:** migraine, hyperhomocysteinemia, nitroglycerine, allodynia, anxiety, photophobia, cortical spreading depression, cortical excitability

## Abstract

Homocysteine is a sulfur-containing endogenous amino acid leading to neurotoxic effects at high concentrations. Population studies suggest an association between plasma homocysteine levels and the risk of migraine headaches. The aim of this study was to analyze the sensitivity of rats with prenatal hyperhomocysteinemia (hHCY) in respect of the development of behavioral correlates of headache and spreading cortical depolarization (CSD) in a migraine model induced by the administration of the nitric oxide (NO) donor nitroglycerin. Animals with hHCY were characterized by migraine-related symptoms such as mechanical hyperalgesia, high-level anxiety, photophobia, as well as an enhanced level of neuronal activity in the somatosensory cortex along with a lower threshold of CSD generation. Likewise, acute or chronic intermittent administration of nitroglycerin also induced the development of mechanical allodynia, photophobia and anxiety in control groups. However, these symptoms were more pronounced in rats with hHCY. Unlike hHCY, nitroglycerin administration did not affect the threshold of CSD generation, but like hHCY, increased the background neuronal activity in layers 2/3 and 4 of the cerebral cortex. The latter was more pronounced in animals with hHCY. Thus, the migraine profile associated with hHCY can be further exaggerated in conditions with enhanced levels of migraine triggering the gaseous transmitter NO. Our data are consistent with the view that high levels of plasma homocysteine can act as a risk factor for the development of migraine.

## 1. Introduction

Homocysteine is a sulfur-containing amino acid produced during the metabolism of methionine. An increase in the concentration of plasma homocysteine, called hyperhomocysteinemia (hHCY), is a pathological condition, which results from impaired metabolism of sulfur-containing amino acids as a result of mutations at genes encoding enzymes, deficiency of coenzymes, namely, folic acid, B12 and B6. Furthermore, environmental factors such as excessive consumption of coffee, alcohol, sedentary lifestyle and intake of certain medications such as L-dihydroxyphenylalanine (L-DOPA) or antiepileptic drugs [1,2,3] contribute to establishing hHCY. An increase in homocysteine levels during prenatal development impairs neurogenesis and plasticity of the developing brain or induces long-term cognitive dysfunctions [4,5,6,7]. In addition, hHCY is a factor in cardiovascular and neurodegenerative diseases [1,8]. A number of population studies indicate the relationship between plasma homocysteine levels and the development of migraine especially migraine with aura [9,10,11]. Moreover, polymorphisms of methylenetetrahydrofolate reductase (MTHFR), a central enzyme in the folate metabolic cycle in which deficiency induces hHCY, were associated with migraine [12]. The possible mechanisms involving hHCY in migraine pathogenesis include endothelial dysfunction, thrombosis [13,14], mitochondrial dysfunction and oxidative stress [15].

The mechanisms of migraine are investigated using various experimental models based on sensitization or stimulation of the trigeminal system [16]. Cortical spreading depression (CSD) is a propagating wave of depolarization of neuronal and glial cells, which induces headache as a result of activation of meningeal nociceptive endings, and is considered an electrophysiological correlate of migraine with aura [17,18]. CSD also has effects on cortical blood flow, initially by an increase predominantly through the release of nitric oxide (NO) followed by long-term oligemia [19]. Systemic administration of the NO donor nitroglycerin (NTG) which causes changes in the neurovascular system is widely used to simulate acute and chronic migraine in humans and animals [20,21,22,23,24,25,26].

In rodents, NTG evokes photophobia, increases meningeal blood flow, and hyperalgesia [27,28], which is prevented by sumatriptan, an anti-migraine drug [27]. In our recent study an increased sensitivity to CSD was demonstrated in rats with hHCY [29], however, the role of neurovascular changes in an NTG-induced migraine model in rats with hHCY, was not investigated.

In the present study, we analyzed mechanical sensitivity, photophobia and the anxiety level in response to acute and chronic NTG administration in rats with prenatal hHCY. In addition, the effects of NTG on the threshold and number of CSD, and neuronal activity in the somatosensory cortex of rats with hHCY were investigated.

## 2. Materials and Methods

### 2.1. The Model of Prenatal Hyperhomocysteinemia

The studies were carried out on male Wistar rats (P45-90) in accordance with the EU Directive 2010/63/EU for animal experiments and the Local Ethics Committee of the Kazan Federal University (KFU) (protocol No. 8 of 5 May 2015, No. 33 of 25 November 2021). The animals were housed in polyethylene cages at room temperature (22 °C) with a 12-h light/dark cycle (lights on at 6 am) and free access to food and water.

The rats in the control groups were born from females (n = 12) receiving a standard diet. Rats with prenatal hHCY were born from females (n = 10) receiving daily methionine (7.7 g/kg diet) with food starting from three weeks before, during pregnancy and during milk feeding [5,6,30,31]. Plasma homocysteine concentration was determined using the Homocysteine Colorimetric Assay Kit (E-BC-K143, ElabScience, Waltham, MA, USA) by spectrophotometry using an ELISA reader (Multiskan FS, Thermo Fisher Scientific, Waltham, MA, USA) [32]. The plasma homocysteine concentration of females on a methionine diet was 17.3 ± 2.3 μM (n = 33), and in control animals—6.3 ± 1.0 μM (n = 12). The homocysteine level in the litter of the control group was 7.4 ± 1.2 µM (n = 12), and in the hHCY group—16.1 ± 0.9 μM (n = 10).

### 2.2. Behavioral Assessment

Background behavioral testing of rats was carried out 2 h before the administration of NTG. The tests were performed in the following order: open field, von Frey’s filaments, followed by the dark-light chamber. Nitroglycerine (NTG, Ozon, Zhigulevsk, Russia, 10 mg/kg in 0.9% NaCl) was injected intraperitoneally (i.p.) [33] and tests were carried out within 1, 2 and 3 h after NTG administration. For chronic experiments, NTG was repetitively administrated every other day for nine days (1, 3, 5, 7, 9 days), in five general NTG injections. Mechanical hypersensitivity was measured two hours before (pre-injection/basal response) and 2 h after (post-injection response) NTG injections [34]. The dark-light chamber test was additionally performed before and after the last injection of NTG on day 9. NaCl was injected as a vehicle in the controls. All behavioral experiments were conducted at the same time and started at about 10.00 am.

#### 2.2.1. Open Field Test

Locomotor behavior and anxiety were measured in open field tests [35] before and during 3 h of acute NTG administration. The open field consists of a square arena of 100 × 100 cm with a wall 36 cm high divided into 25 squares of 20 × 20 cm (Open Science, Moscow, Russia) equipped with a video system Sony SSC-G118 (Japan) and an illumination of ∼175 lux. The animal was placed in the middle of the open field for 3 min and the total of line crossings (i.e., horizontal activity), activity in the central area (central square) and peripheral area or thigmotaxis (16 squares along the walls), rearing and grooming episodes were assessed by an independent researcher [36,37].

After each trial, the open field was cleaned with 75% ethanol and permitted to dry between tests.

#### 2.2.2. Von Frey Test

Mechanical sensitivity was assessed with a series of calibrated Von Frey filaments (Ugo Basile, Gemonio, Italy) with 0.008 to 8 g of the target force, which corresponds to 2.53–61.7 g/mm^2^ pressure. 30 min before the test, the rat was placed in an individual transparent box with a mesh floor.

The mechanical withdrawal thresholds were determined according to the up-and-down method [38,39]. von Frey filaments were applied by approaching the plantar surface of the paw from the underside of the mesh stand. We always started by testing with a 0.4 g (3.61 g/mm^2^) filament. In all cases, the tip of the filament was pressed against the plantar surface of one hind paw maintained for 1–3 s. A response is defined as withdrawal, shaking or licking of the paw. In the absence of a response, a heavier filament (up) was tried after 10 s, and in the presence of a response, a lighter filament (down) was tested. This pattern was followed for a maximum of 4 filaments following the first response.

#### 2.2.3. Light/Dark Transition Test

The light-dark box test was used to measure anxiety-like behavior [40]. The light–dark box consists of two equally-sized chambers-one illuminated (150 lux) and one darkened (1–2 lux) 40 × 20 × 40 cm/each connected with a passageway 7 × 7 cm (Open Science, Moscow, Russia) equipped with a video system Sony SSC-G118 (Tokyo, Japan). The rats were placed in the light compartment and allowed to explore the apparatus for 3 min. We measured the latency to enter the dark chamber and the time spent in the light chamber.

### 2.3. Electrophysiological Recordings of Cortical Spreading Depression

Preparation of the animals for electrophysiological experiments was performed under isoflurane (Baxter, Deerfield, IL, USA) anesthesia (5% induction and 1.5–2% for maintenance) as previously described [29]. The skin and tissue were removed from the head. Urethane (1.5 g/kg i.p.) was injected by the end of surgery and the animal was fixed by the head to the frame of a stereotaxic apparatus by the attached bars. Two 2 mm diameter holes were drilled on the left side: one above the somatosensory cortex for electrophysiological recordings (3.5 mm posterior and 4 mm lateral to bregma) and the second for the chemical induction of CSD (8 mm posterior and 4 mm lateral to the bregma). A chlorinated silver wire placed in the cerebellum, or the visual cortex served as a ground electrode. The body temperature was maintained at 37 ± 0.5 °C using a self-regulating heating pad (TCAT-2LV controller, Physitemp Instruments INC, Clifton, NJ, USA).

Cortical spreading depression (CSD) and multiple-unit activity (MUA) were recorded by a 16-sites linear silicon probe (Neuronexus Technologies, Ann Arbor, MI, USA), 100 μm between recording sites, in direct current mode (input range = ± 131 mV) using DigitalLynx (Neuralynx, Bozeman, MO, USA) (band-pass range 0–9000 Hz) [29,41]. The record was digitized at 32 kHz and was analyzed in the MATLAB environment.

The probe was inserted in the cortex to a depth of 1700 ± 100 µm. CSD was initiated by application of KCl solution (10 µL) at increasing concentrations (0.01, 0.05, 0. 1, 0.3, 0.6 and 1 M) into the second hole. After application, we observed the appearance of CSD for 10 min. If CSD did not occur, KCl was removed and the hole was flushed with artificial cerebrospinal fluid (for 20 min, then KCl was applied at a higher concentration [29,42].

CSD recordings consisted of depolarization waves that spread along the layers of the cortical column. Detection of CSD was carried out visually according to the characteristic form of the local field potential (LFP). The baseline level of LFP was calculated for each channel in the time window between 20 and 10 s preceding CSD. For CSD detection the signals were filtered at low frequencies with a threshold of <3 Hz, and the onset of CSD on each channel was calculated from the time of the maximum negative peak of the first derivative of the LFP. CSDs were considered as events with amplitudes no less than 5 mV and durations of more than 6 s [29]. In each experiment, the minimal concentration of KCl evoking the CSD and the number and duration of CSDs were calculated in response to each concentration of KCl.

For multiple unit activity (MUA) detection, the wide-band signal was filtered (bandpass 300–3000 Hz) and negative events exceeding five standard deviations in amplitude were considered as action potentials. The spike frequency was analyzed before and after nitroglycerine injection (10 µg/kg ip). Application of KCl was carried out 1 h after injection of nitroglycerine.

To determine the time of recovery of neuronal activity after CSD, MUA was analyzed in L4 during the 10 min before, and 10 min after KCl application.

### 2.4. Statistical Analysis

The processing of experimental data was performed using specially developed software based on MATLAB—ExpressAnalysis and Eview (Andrey Zakharov, https://github.com/AndreyZakharovExp (accessed on 1 January 2015)). The normality of the sample data was evaluated with the Kolmogorov–Smirnov test (OriginLab Corp, Northampton, MA, USA). Mann–Whitney test was used for comparison of independent samples and the Wilcoxon test for related samples. Differences were considered statistically significant at *p* < 0.05. All results are presented as M ± m, where M is the mean, and m is the error of the mean.

## 3. Results

### 3.1. Effects of Acute and Chronic Nitroglycerine (NTG) Administration on Mechanical Sensitivity of Rats with hHCY

**Figure 1:**
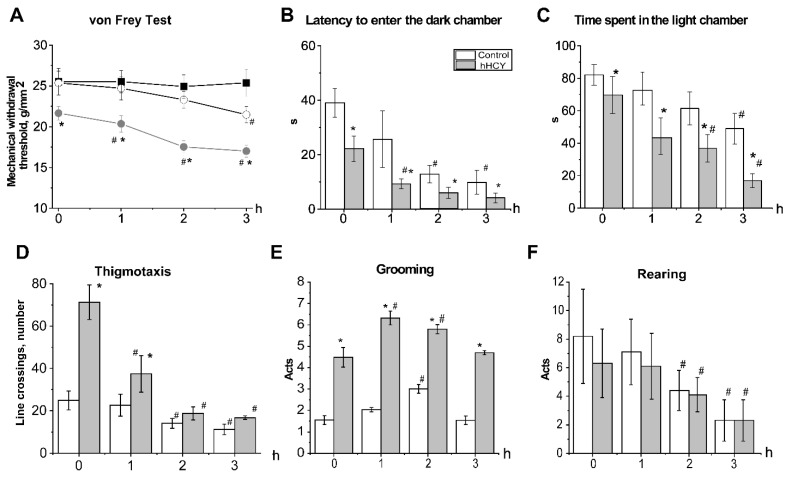
Effects of acute nitroglycerine (NTG) administration on mechanical sensitivity and behavior in the light-dark transition test and open field. (**A**) Mechanical withdrawal threshold of the plantar surface of the hind paw of rats, control (open circle), hHCY group (grey circle) and vehicle (black square) groups before (0) and during 3 h after NTG administration. (**B**) Latency to enter the dark chamber and (**C**) time spent in the light chamber, of rats from the control (white column) and hHCY (grey column) groups before (0) and during 3 h of NTG administration. The number of line crossings in the peripheral area of the open field or thigmotaxis (**D**), grooming acts (**E**) and rearing (**F**) of rats from control (white columns) and hHCY groups (grey columns) before (0) and during three hours of NTG administration. * *p* < 0.05 compared to the control group, # *p* < 0.01 compared to the initial values.

The initial threshold of mechanical sensitivity in the control group was 25.3 ± 1.4 g/mm^2^ (n = 10). In the hHCY group (n = 10) it was significantly lower-21.6 ± 0.8 g/mm^2^ (*p* < 0.05) (Figure 1A). Administration of NTG decreased the mechanical threshold of animals from the hHCY group during the first hour which achieved a minimal level by the second hour of observation of 17.5 ± 0.7 g/mm^2^ (*p* < 0.05, Figure 1A). In the control group mechanical hyperalgesia was developed only by the third hour of NTG injection (21.4 ± 1.1 g/mm^2^ *p* < 0.05, Figure 1A). NaCl administration did not change the mechanical sensitivity (Figure 1A).

**Figure 2:**
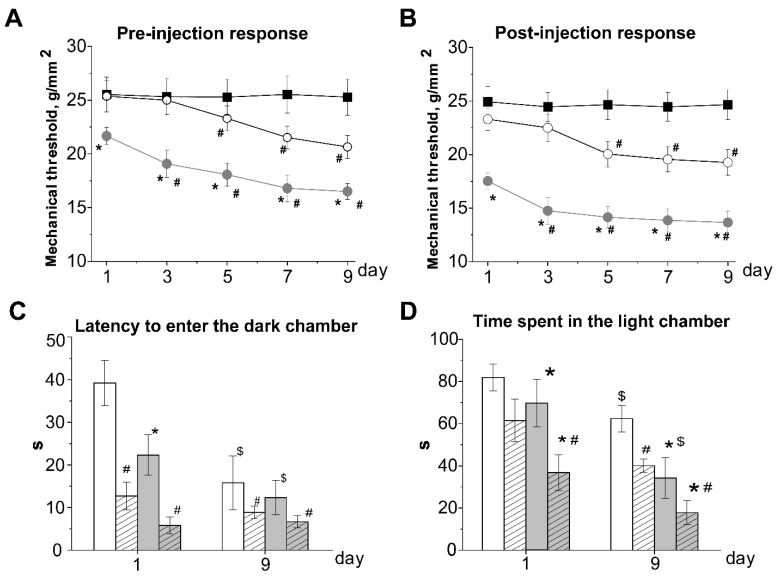
Effects of chronic nitroglycerine (NTG) administration on mechanical hyperalgesia and behavior in the light-dark transition test. Mechanical withdrawal thresholds of rats from the control (open circle), hHCY groups (grey circle) and vehicle (black square) groups before (**A**), basal response) and two hours after (**B**), post-injection response) chronic NTG administration. * *p* < 0.05 compared to the control group, # *p* < 0.05 compared to the initial values. Latency to enter the dark chamber (**C**) and time the rats spent in the light chamber (**D**) before (open columns) and two hours after (dashed columns) NTG administration at the first and 9th day of experimentation; control (white column) and hHCY (grey dashed column) groups, * *p* < 0.05 compared to the control group, # *p* < 0.05 compared to the pre-injection level, $ *p* < 0.05 compared to values of the first day.

We further tested mechanical thresholds before and 2 h after NTG administration every second day for 9 days, resulting in a total of 5 NTG injections. Repetitive NTG administration produced a significant chronic basal mechanical hypersensitivity assessed by testing prior to each administration of NTG-pre-injection basal response (Figure 2A). In the control group significant basal allodynia was observed on the 5th day of the experiment (23.3 ± 1.1 g/mm^2^, *p* < 0.05, Figure 2A). In rats from the hHCY group, the initial threshold level was lower compared to the control, and a further decrease was observed after the first injection on the third day of the experiment (19.1 ± 1.2 g/mm^2^, *p* < 0.05, Figure 2A). NTG induced acute mechanical hyperalgesia-post-injection response 2 h after the third NTG injection on day 5 of the chronic experiment was observed in the control group (Figure 2B). In rats from hHCY group post-injection, mechanical hyperalgesia was observed after the first injection of NTG (Figure 2B).

### 3.2. Anxiety and Photophobia in Rats with hHCY after Administration of Nitroglycerine in the Light-Dark Transition Test

The initial parameters recorded in the dark-light chamber test were different between the animals of the two groups. The time to the first entry into the dark chamber in the control group was 39.1 ± 5.3 s (n = 16), and 22.3 ± 4.7 s—and in the hHCY group (n = 10, *p* < 0.05, Figure 1 B). Administration of NTG reduced the latency to enter the dark chamber during the second hour of nitroglycerine action in the control group (12.7 ± 3.3 s, *p* < 0.05) and within the first hour—in the hHCY group (9.3 ± 1.8 s, *p* < 0.05) (Figure 1B). The total time spent in the light chamber was significantly longer for animals from the control group (81.9 ± 6.4 s) compared to the hHCY group (69.7 ± 11.3 s, n=10, *p* < 0.05, Figure 1C). During the second hour, this time was reduced in both groups to 61.4 ± 10.1 s in the control and 36.8 ± 8.5 s—in the hHCY group (*p* < 0.05) (Figure 1C).

Testing in the light-dark chamber was additionally performed before and two hours after the fifth NTG injection on the ninth day of repetitive NTG administration (Figure 2C,D). In the vehicle group latency to the first entrance into the dark chamber and time spent in the light chamber did not change on the ninth day of testing. The basal latency of the first entrance into the dark chamber before the last injection of NTG was lower compared to the first day, both in control and hHCY groups and was 15.8 ± 6.3 s and 12.3 ± 4.0 s (*p* > 0.05), respectively (Figure 2C). After two hours of NTG injection this parameter further decreased to 8.8 ± 1.4 s and 6.6 ± 1.4 s (*p* > 0.05, Figure 2C). The time spent in the light chamber before the last injection was lower compared to the first day in both groups and was 62.3 ± 6.1 s in the control and 34.2 ± 9.7 s in the hHCY group (*p* < 0.05) (Figure 2D). Two hours after NTG injection this time further decreased and was 40.1 ± 3.2 s in the control and 17.8 ± 5.7 s in the hHCY groups (*p* < 0.05, Figure 2 D). NaCl injection did not change any parameters of dark-light chamber tests in the vehicle group during the first and on the 9th day of experimentation.

### 3.3. Effects of Acute Nitroglycerine Administration on the Behavior of Rats with hHCY in the Open Field

Initial behavioral reactions in the open field were different in animals from both groups. The line crossings were significantly higher in animals of the hHCY group (80.1 ± 6.8, *p* < 0.05 versus 33.7 ± 5.2 in control, Table 1). At the same time rats with hHCY spent more time in the peripheral area compared to the central zone of the open field (Table 1, Figure 1D).

Rearing did not differ significantly between the groups (Figure 1 F). The level of grooming was higher in rats of the hHCY (4.5 ± 0.4 acts) compared to the control group (1.5 ± 0.2 acts *p* < 0.05, Figure 1E). One hour after injection, of NTG the horizontal activity decreased significantly in animals from the hHCY group and achieved a minimum level during the second hour (Table 1). Line crossings of rats from the control group significantly decreased only during the second hour of NTG action (Table 1). Similar changes were observed in the activity in the central and peripheral areas (Table 1, Figure 1D). An increase in grooming acts was observed in animals of the hHCY group during the first hour of NTG action, whereas in the control group it increased only during the second hour of NTG action (Figure 1E). Rearing significantly reduced in both groups during the second hour after NTG administration (Figure 1F).

### 3.4. Effects of Nitroglycerine Administration on CSD and MUA in Rats with hHCY

**Figure 3:**
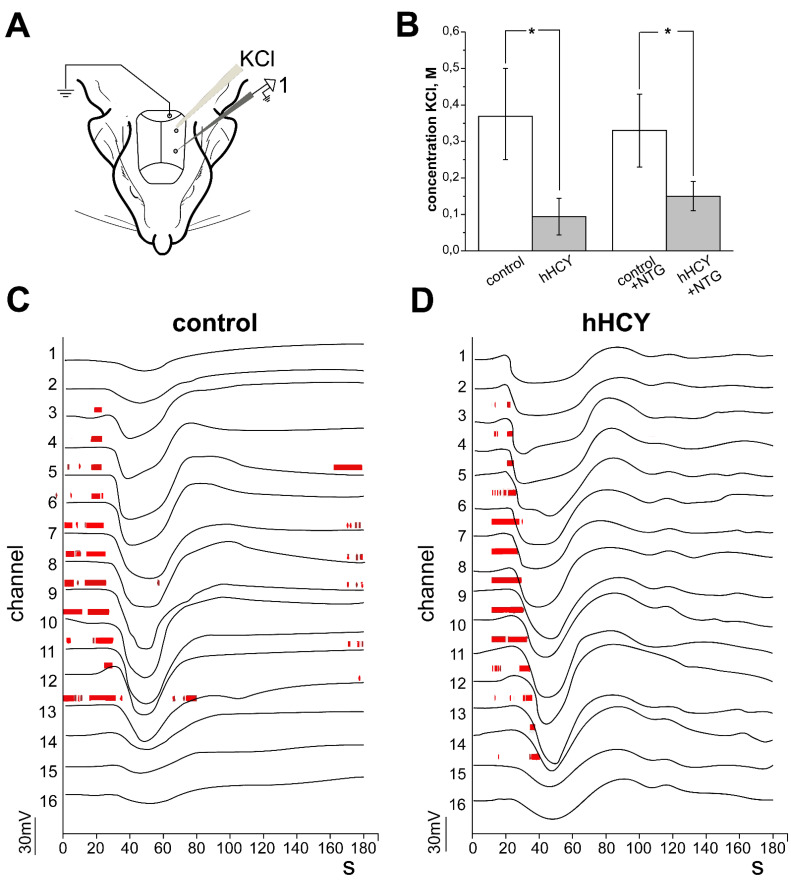
In vivo recording of cortical spreading depression (CSD) induced by KCl application and effects of nitroglycerine (NTG) on the threshold of CSD. (**A**) Schematic representation of the electrophysiological experiment. (**B**) The minimum KCl concentration inducing CSD before and after the administration of nitroglycerine in rats of the control (white columns) and hHCY (gray columns) groups. (**C**,**D**) Examples of KCl induced CSD recorded by a 16-site linear silicon probe (100 μm distances between recording sites) in all layers of cortical columns of control (**C**) and hHCY (**D**) groups. The multiple unit activity (MUA) marked by vertical red lines were detected as negative events exceeding eight standard deviations in amplitude, * *p* < 0.05 compared to control group.

As previously reported the minimum threshold concentration of KCl for eliciting CSD was lower in animals of the hHCY group (Figure 3B) [29]. In controls the threshold concentration of KCl was 0.37 ± 0.13 M (n = 7) and in the hHCY group 0.09 ± 0.05 M (n = 6, *p* < 0.05). Administration of NTG did not change the threshold of the KCl concentration in both groups—(0.33 ± 0.17 M in controls (n = 7) and 0.15 ± 0.04 M (n = 6) in the hHCY group, Figure 3B).

**Figure 4:**
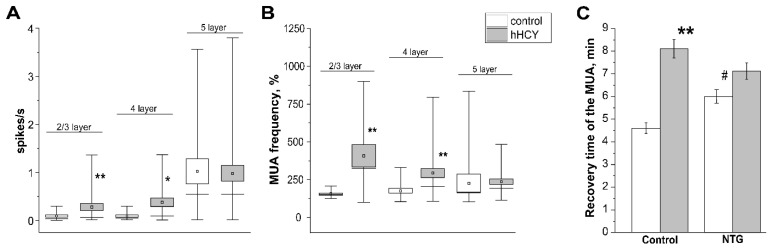
Effects of nitroglycerine (NTG) on MUA in rats of control and hHCY groups. (**A**) Background MUA frequency before the administration of NTG in animals of the control (white boxplots) and hHCY (grey boxplots) groups, (**B**) increase in MUA (in %) after administration of NTG in animals of the control (white boxplots) and hHCY (grey boxplots) groups, (**C**) recovery time of MUA after CSD wave before and after injections of NTG in control (white columns) and hHCY (grey columns) groups. * *p* < 0.05, ** *p* < 0.01 compared to control, # *p* < 0.05 compared to initial values.

The number of CSDs and duration of CSD occurrence elevated with an increase in the KCl concentration in both groups (Table 2), and the introduction of NTG did not affect these parameters. As another independent indicator of neuronal excitability, we analyzed MUA in all layers of the somatosensory cortex before and after the administration of NTG. Analysis of the background MUA revealed a high activity of neurons in the supragranular (layer 2/3) and granular (layer 4) layers of rats with hHCY (Figure 4A): 0.09 ± 0.02 s^−1^ in control and 0.28 ± 0.07 s^−1^ in hHCY (*p* < 0.01) in layer 2/3; 0.11 ± 0.02 s^−1^ in control and 0.38 ± 0.08 s^−1^ in hHCY (*p* < 0.05) in layer 4; 1.02 ± 0.26 s^−1^ in control and 0.98 ± 0.16 s^−1^ in hHCY in layer 5 (*p* > 0.05, Figure 4A).

One hour after NTG injection, an increase in the frequency of MUA was observed in all cortical layers with a higher increase in rats of the hHCY group. In the layer 2/3 MUA frequency increased up to 155.34 ± 7.25% (n = 7) and in the control up to 407 ± 74.83% (n = 6, *p* < 0.01). In layer 4 MUA increased up to 146 ± 16.66% in control and 293.29 ± 29.63% in hHCY groups (*p* < 0.01); in layer 5—up to 225.14 ± 62.39% in control and 236.71 ± 18.62% in hHCY groups (*p* > 0.05, Figure 4B).

During the generation of the CSD wave, a complete absence of neuronal activity was observed both in control rats and in animals of the hHCY group. However, after 5-10 min, the MUA frequency was slowly restored [43,44]. In the control group MUA after CSD recovered after 4.6 ± 0.2 min (n = 5) D, and in the hHCY group after 8.1 ± 0.5 min (n = 7, *p* < 0.01). NTG administration increased the recovery time to 6.0 ± 0.3 min (n = 7, *p* < 0.05) in the control group without a significant effect in the hHCY group (7.12 ± 0.5 s, n = 6, Figure 4C).

## 4. Discussion

In this study, for the first time, we combined in rats, two migraine-promoting factors: the NO donor NTG and high levels of endogenous amino acid homocysteine leading to the syndrome of hHCY, to characterize the behavioral changes along with electrophysiological parameters of cortical excitability. Our main findings are that the combination of these two clinically relevant migraine-related factors results in a complex algesic phenotype including higher photophobia, anxiety and mechanical hyperalgesia as well as enhanced neuronal activity in some layers of the cerebral cortex.

Migraine is a neurovascular disease characterized by regular headache attacks accompanied by various symptoms such as blurred vision, nausea and vomiting, as well as sensitivity to light (photophobia) or sound stimuli [45]. Moreover, various autonomic and emotional disturbances, like depression and anxiety are often observed during migraine development [46,47]. In about one-third of patients, migraine attacks are preceded by an aura consisting of visual, sensory, speech or cognitive impairments associated with the development of cortical spreading depression (CSD) in the respective cortical areas [48].

Clinical studies suggest a relationship between plasma homocysteine levels and the development of migraine [9,10,11,49,50]. However, experimental evidence on the mechanistical link between hHCY and migraine was missing. In this study, we used a model of prenatal hHCY in rats, where a high level of homocysteine was induced in females by feeding them with a high methionine diet [5,6,31]. In rats born from females with hHCY, a high level of homocysteine in plasma remained even without the additional introduction of methionine into the diet, which is probably associated with a violation of the metabolic methionine/homocysteine cycle during the perinatal period [6].

One of the known triggers of migraine is NO, which evokes direct stimulation of the trigeminovascular system at the vascular level, as well as the ganglia and caudal nucleus of the trigeminal nerve, resulting in peripheral and central sensitization [51,52,53]. It has been shown that CSD induces the release of endogenous NO, which has pro-nociceptive effects through the activation of guanylate cyclase and the release of the calcitonin-related gene peptide (CGRP) from trigeminal ganglion neurons [54,55]. The participation of endogenous NO production in the pathogenesis of migraine is confirmed by the anti-migraine effects of the NO-synthases inhibitor - N-monoethyl-L-arginine (L-NMMA) [56]. Injection of NTG, a NO donor, is a common experimental model of migraine-related headache in rodents, characterized by mechanical allodynia, photophobia and vasodilation of meningeal vessels [24,28].

One of the migraine symptoms is mechanical allodynia, which is observed not only in the area of innervation of the trigeminal nerve in the head, but also in other parts of the body [27]. We found that acute administration of NTG decreased the mechanical threshold of the hind paw in the control group only after 3 h. In the hHCY group, the background mechanical sensitivity was higher as was shown previously by our group [29,57] and NTG further decreased the mechanical threshold at 1 h after injection. Chronic intermittent NTG administration induced basal allodynia and post-injection responses by the 5th day of the experiment in the control group similar to previous data [58,59,60] which appears to reflect the development of central sensitization of nociceptive pathways specific for chronic migraine [25,27,40,61,62].

In the hHCY group basal allodynia and acute mechanical hyperalgesia aggravated during repetitive NTG injections beginning from the first day of observation. Similar results consistent with migraine phenotype were obtained in the light/ dark transition test, where the initial level of anxiety and photophobia was higher in rats of the hHCY group. Acute NTG administration reduced the time of the first entry into the dark chamber and the total time spent in the light chamber in both groups, however, these indicators of photophobia and anxiety developed faster in rats with hHCY. On the 9th day of chronic NTG injections, the basal values of the first entry into the dark chamber and the total time spent in the light chamber were lower compared to the 1st experimental day in both groups and further decreased after the last NTG injection. In hHCY rats, time spent in the light chamber was shorter compared to the control group. Photophobia and reduced motor activity in rats were shown previously during repetitive NTG administration [63].

Behavioral tests in the open field during acute NTG administration allowed us to assess the total locomotor activity and anxiety level [37,41,64]. Rats with hHCY demonstrated hyperactivity in the open field which may be explained by cortex excitability and similar observations were reported in prenatal and postnatal models of hHCY [65,66,67]. At the same time activity in the central area was lower and thigmotaxis was higher in rats with hHCY which along with increased grooming suggests a higher level of anxiety [40,68] similar to previous data obtained from the offspring born from females with hHCY and in a postnatal model of hHCY in adult rats [6,69,70,71].

NTG reduced horizontal activity and increased thigmotaxis in both groups, however, in the hHCY group significant changes were observed during the first hour after injection, whereas in the control group only during the second hour. Similarly, the level of grooming increased in the animals of the hHCY group within the first hour after the injection of NTG, while in the control group this effect was observed only in the second hour. Therefore, rats with hHCY showed higher sensitivity to behavioral signs of headache in acute and chronic NTG-induced models of migraine in rodents.

Next, we analyzed the effects of NTG on the sensitivity to CSD, which characterizes the excitability of the cortex in rats with hHCY. Experimental and clinical evidence suggests that CSD is the main trigger of migraine attacks in the type of migraine with aura and is related to aura symptoms [72]. CSD was induced by applying KCl to the dura mater of the rat brain which constitutes a common approach for modeling this cortical migraine-related phenomenon [17,18]. In rats with hHCY, the threshold concentration of KCl for CSD generation was lower compared to the control group along with a higher background neuronal activity, which is consistent with our previous studies [29] and epidemiological data on the correlation of homocysteine levels and migraine with aura in humans [9,73].

The injection of NTG did not change the threshold of CSD generation as well as the number and duration of CSD in both groups, which is consistent with literature data in the absence of the effect of exogenous NO on the threshold and parameters of CSD [27,40,74]. However, it has been shown that NTG increases the velocity of CSD propagation [27,74], while the inhibition of NO synthase reduces the threshold for CSD generation [75]. The latter indicates a role of endogenous NO in the regulation of ionic homeostasis and the excitability of cortical neurons [76]. Indeed, NTG in our experiments, increased MUA frequency in all layers of the cortical column in both groups, with a higher increase in the supragranular and granular layers of the cerebral cortex of the hHCY group. These effects may be mediated both by a direct effect of NO on neurons in the cerebral cortex and sensitization of peripheral afferents of the trigeminal nerve that innervate the dura mater [54]. Guanylate cyclase, which produces cGMP with subsequent activation of protein kinase G, is one of the main targets of NO [77,78]. In the presynaptic regions, protein kinase G phosphorylates synaptophysin, which is necessary for the fusion of glutamate-containing vesicles with the presynaptic membrane, which enhances glutamatergic transmission [78]. In addition, direct and cGMP-mediated effects of NO on ion channels and neuronal excitability have been shown [77,79].

Therefore, along with higher sensitivity to the development of CSD hHCY aggravates behavioral changes in the NTG-induced acute and chronic models of migraine which may be associated with a number of factors leading to sensitization of the peripheral and central structures involved in the generation of headache-related migraine. It has been shown that chronically elevated homocysteine concentrations induced oxidative stress and increased the level of pro-inflammatory cytokines in brain tissues [5,6,80], which was shown to trigger inflammation and impact the NTG model of chronic migraine [60]. Due to oxidative stress and inflammation in hHCY reduced NO bioavailability results in endothelial dysfunctions characterized by increased vascular tone, thrombosis and vascular permeability [55,81]. Moreover, homocysteine is a potent excitatory aminoacid, and may induce upregulation of NMDA receptors or enhanced sensitivity to glutamate underlying hyperexcitability in hHCY conditions [82,83].

The obtained results suggest that rats with hHCY are characterized by both increased excitability of neurons in the cerebral cortex and higher sensitivity of peripheral afferents of the trigeminal nerve, innervating the dura mater. Accordingly, careful control, lowering of homocysteine level and neutralization of hHCY consequences may have positive effects on the frequency and severity of migraine attacks. Indeed, clinical studies showed that vitamin B supplementation reduced plasma homocysteine levels and the frequency of attacks [84,85,86].

## 5. Conclusions

In conclusion, a chronic increase in the level of homocysteine in rats leads to allodynia, photophobia, anxiety, as well as to increased neuronal activity in the somatosensory cortex along with higher susceptibility for CSD generation, which correlates migraine with aura. Moreover, in the model of acute and chronic NTG-induced migraine, rats with prenatal hHCY showed greater sensitivity to the development of photophobia, anxiety and hyperalgesia. At the same time, the NO donor NTG did not affect the threshold of CSD generation but increased the background activity of neurons in 2/3 and 4 layers of the cerebral cortex, more significantly in animals with hHCY. It is suggested that a high plasma concentration of homocysteine can serve as a risk factor for the development of migraine headaches and constitutes a prognostic factor for assessing the severity of attacks, and a decrease in its level can facilitate the course of the disease.

## Figures and Tables

**Table 1:** Locomotor activity of rats in the open field test before and after NTG administration.

	Control (Mean ± SEM)	Hhcy (Mean ± SEM)
**Total number of line crossings**		
Before NTG	33.7 ± 5.2	80.1 ± 6.8 *
+NTG 1h	26.1 ± 5.6	40.7 ± 7.8 #*
+NTG 2h	21.1 ± 4.9 #	23.5 ± 2.6 #
+NTG 3h	12.3 ± 2.3 #	19.7 ± 0.4 #
**Central zone activity**		
Before NTG	6.5 ± 1.2	3.1 ± 0.7 *
+NTG 1h	3.2 ± 0.6 #	2.2 ± 0.6 #*
+NTG 2h	1.4 ± 0.2 #*	0.8 ± 0.3 #
+NTG 3h	0.8 ± 0.2 #	0.4 ± 0.2 #
**Thigmotaxis**		
Before NTG	24.8 ± 4.4	71.3 ± 8.1 *
+NTG 1h	22.6 ± 5.2	37.4 ± 8.6 #*
+NTG 2h	14.1 ± 2.3 #	18.7 ± 3.1 #
+NTG 3h	11.2 ± 2.4 #	16.7 ± 0.8 #

* *p* < 0.05 compared to control; # *p* < 0.05 compared to initial value.

**Table 2:** Effect of NTG on parameters of CSD in rats from control and hHCY groups.

KCl concentration	Number of Animals with CSD/all Animals	Number of CSD (Mean ± SEM)	Duration of CSD Occurrence (min) (Mean ± SEM)
**0.05 M**			
Control	1/7	1 ± 0	--
hHCY	3/6	1 ± 0	--
Control + NTG	1/5	1 ± 0	--
hHCY + NTG	2/7	1 ± 0	--
**0.1 M**			
Control	3/7	2.1 ± 0.4	5.4 ± 1.9
hHCY	5/6	2.4 ± 0.3	10.4 ± 1.6
Control + NTG	2/5	2.0 ± 1.0	6.4 ± 3.1
hHCY + NTG	5/7	2.2 ± 0.7	9.1 ± 3.4
**0.2 M**			
Control	4/7	4.1 ± 0.6	13.3 ± 1.5
hHCY	6/6	4.7 ± 1.0	17.6 ± 3.0
Control + NTG	3/5	2.6 ± 0.8	7.4 ± 4.2
hHCY + NTG	5/7	3.4 ± 1.0	9.0 ± 3.4
**0.3 M**			
Control	4/7	3.9 ± 0.7	10.2 ± 3.8
hHCY	6/6	3.6 ± 0.7	12.3 ± 3.0
Control + NTG	4/5	2.7 ± 0.8	10.6 ± 4.8
hHCY + NTG	7/7	1.8 ± 0.4	6.4 ± 1.8
**0.6 M**			
Control	6/7	5.9 ± 1.2	19.3 ± 2.3
hHCY	6/6	6.4 ± 1.5	23.2 ± 8.2
Control + NTG	4/5	5.5 ± 1.5	20.4 ± 5.2
hHCY + NTG	7/7	3.5 ± 0.9	14.6 ± 2.6
**1 M**			
Control	7/7	7.2 ± 1.3	25.6 ± 4.5
hHCY	6/6	6.8 ± 1.7	20.3 ± 6.3
Control + NTG	5/5	5.4 ± 2.5	20.1 ± 6.0
hHCY + NTG	7/7	4.1 ± 0.8	14.2 ± 2.3

## Abbreviations

hHCY	hyperhomocysteinemia
CSD	cortical spreading depression
MUA	multiple unit activity
MTHFR	methylenetetrahyrdofolate reductase
LFP	local field potential
NTG	nitroglycerine
